# Selenium nanoparticles as anti-infective implant coatings for trauma orthopedics against methicillin-resistant *Staphylococcus aureus* and *epidermidis*: in vitro and in vivo assessment

**DOI:** 10.2147/IJN.S197737

**Published:** 2019-07-01

**Authors:** Phong A Tran, Neil O'Brien-Simpson, Jason A Palmer, Nathalie Bock, Eric C Reynolds, Thomas J Webster, Anand Deva, Wayne A Morrison, Andrea J O'Connor

**Affiliations:** 1 School of Chemistry, Physics and Mechanical Engeneering, Faculty of Science and Engeneering, Queensland University of Technology (QUT), Brisbane, Queensland 4000, Australia; 2 Interface Science and Materials Engineering Group, School of Chemistry, Physics & Mechanical Engineering, QUT, Brisbane, Queensland 4000, Australia; 3 Departments of Chemical and Biomedical Engineering, The Particulate Fluid Processing Centre, The University of Melbourne, Melbourne, Victoria 3010, Australia; 4 Oral Health Cooperative Research Centre, Melbourne Dental School, The University of Melbourne, Melbourne, Victoria 3010, Australia; 5 O’ Brien Institute, St. Vincent’s Institute of Medical Research, Fitzroy, Victoria 3065, Australia; 6 School of Biomedical Sciences, Faculty of Health, Institute of Health and Biomedical Innovation, Translational Research Institute, QUT, Brisbane, QLD, Australia; 7 Department of Chemical Engineering, Northeastern University, Boston, MA 02115, USA; 8 Surgical Infection Research Group, Australian School of Advanced Medicine, Macquarie University, Sydney, NSW, Australia

**Keywords:** orthopedic, implants, antimicrobial, biofilm, selenium, nanoparticles

## Abstract

**Background:** Bacterial infection is a common and serious complication in orthopedic implants following traumatic injury, which is often associated with extensive soft tissue damage and contaminated wounds. Multidrug-resistant bacteria have been found in these infected wounds, especially in patients who have multi trauma and prolonged stay in intensive care units.Purpose: The objective of this study was to develop a coating on orthopedic implants that is effective against drug-resistant bacteria.

**Methods and results:** We applied nanoparticles (30-70nm) of the trace element selenium (Se) as a coating through surface-induced nucleation-deposition on titanium implants and investigated the antimicrobial activity against drug resistant bacteria including Methicillin-resistant *Staphylococcus aureus* (MRSA) and Methicillin-resistant *Staphylococcus epidermidis* (MRSE) in vitro and in an infected femur model in rats.The nanoparticles were shown in vitro to have antimicrobial activity at concentrations as low as 0.5ppm. The nanoparticle coatings strongly inhibited biofilm formation on the implants and reduced the number of viable bacteria in the surrounding tissue following inoculation of implants with biofilm forming doses of bacteria.

**Conclusion:** This study shows a proof of concept for a selenium nanoparticle coatings as a potential anti-infective barrier for orthopedic medical devices in the setting of contamination with multi-resistant bacteria. It also represents one of the few (if only) in vivo assessment of selenium nanoparticle coatings on reducing antibiotic-resistant orthopedic implant infections.

## Background

Infection is a leading complication following implantation of fixation devices for traumatic orthopedic injuries.^1^–^4^ Traumatic wounds, such as those resulting from accidents, military combat and/or training, often involve extensive tissue damage in highly contaminated environments.^5^ Effective infection control is known to be key in the healing and rapid recovery of injuries in such cases.^6^ Despite clinically effective debridement and antibiotic treatment for these injuries, the infection rates remain as high as 15% and 20% in traumatic extremity injuries and abdominal wounds, respectively.^7^,^8^ The introduction of a medical device to treat the wound and/or restore anatomic integrity can provide much needed benefits but comes with the risk of infection. Additionally, the relative immunosuppression following trauma may potentiate the growth of either contaminating bacteria or hospital-acquired infection. Multidrug-resistant bacteria such as *Acinetobacter baumannii, Pseudomonas aeruginosa*, extended-spectrum β-lactamase-producing *Klebsiella* species, *Escherichia coli*, and methicillin-resistant *Staphylococcus* strains have been found in these infected wounds,^7^,^8^ especially in patients who have multitrauma and prolonged stay in intensive care units. The presence of medical devices has been associated with the formation of biofilms (ie, bacteria-containing polysaccharide matrices that are extremely resistant to host defenses and antibiotic treatment).^9^–^11^ Biofilm-related device-associated infections have been shown to be a cause of both implant failure, reoperation, and even death.^12^,^13^

In the last few decades, new strategies have been developed to tackle the problems of bacterial drug resistance and biofilm formation. These strategies can be roughly divided into two categories: controlled delivery of antibiotics and development of non-drug antimicrobial materials. The antibiotic delivery approach aims to specifically target high doses of drug molecules to the infection sites, limiting sub-lethal doses and/or non-specific exposure of bacteria to the drugs to combat further development of antibiotic resistance.^14^–^16^ Delivery strategies to improve drug penetration into bacterial biofilms are also being actively investigated.^16^ In the second category, new antimicrobial agents which do not contain antibiotics are being developed. Examples of this include metallic/ceramic nanoparticles (NPs) (such as silver, copper oxide, zinc oxide, gold, titanium dioxide, etc.),^17^–^20^ polymeric materials (such as chitosan^21^), quaternary ammonium compounds,^22^,^23^ and antimicrobial peptides.^24^ Surface modification to repel bacteria or kill on contact is also a promising approach.^25^,^26^ Nano-topography has been shown by a number of groups to reduce bacteria adhesion and damage the bacteria cell membrane (for a review, refer to^27^ and^28^). Negatively charged surfaces or protein-repelling polymer coatings, such as poly(ethylene oxide) (PEO) brushes, were also shown to prevent bacterial adhesion.^25^,^29^ A number of polyatomic polymers are bactericidal, and their immobilization onto surfaces has rendered antimicrobial activities (for an extensive review, refer to^30^).

Recently, selenium nanoparticles (Se NPs) – a trace, essential, metalloid element – have appeared as a promising antimicrobial material in both suspension and immobilized forms.^31^–^34^ Importantly, these particles were shown to have very low toxicity to mammalian cells, making them an attractive antimicrobial agent.^35^–^38^ In our laboratories, we have immobilized Se NPs on polymer surfaces and demonstrated their in vitro antimicrobial activities.^31^,^32^

In this current study, we aimed to investigate the antimicrobial activities of these NPs immobilized on metal surface against methicillin-resistant *Staphylococcus aureus* (MRSA) and *Staphylococcus epidermidis* (MRSE), two key drug-resistant bacteria in nosocomial infections and trauma orthopedic surgery. In vitro characterization experiments of the Se particle performance against these bacteria were followed by pilot in vivo trials in a rat femur model inoculated with bacteria at the time of surgery.

## Methods

### NP synthesis and coating on titanium plates and screws

Sodium selenite, L-ascorbic acid, and polyvinyl alcohol (PVA) were obtained from Sigma Aldrich (Castle Hill, NSW, Australia) and dissolved in purified water (Millipore, Bayswater, VIC, Australia 18 MΩ/cm) to concentrations of 10 mM, 100 mM, and 20 mg/mL, respectively. For aqueous phase synthesis of PVA-stabilized Se NPs at room temperature, sodium selenite and PVA solutions were mixed at volume ratios of 1:1. An ascorbic acid solution was then added to this solution at a volume ratio of 3:20 (PVA+sodium selenite: ascorbic acid). The synthesis solution was mixed using magnetic stirrers and left at room temperature for 2 hrs for complete reduction of selenite by ascorbic acid to produce elemental Se. The reaction resulted in a solution color change from clear white to clear red which was detected using spectrophotometry (Varian Cary 50MPR, Agilent Technologies, Santa Clara, CA, USA). The particles were then washed several times with Millipore water by centrifugation at 10,000 rpm (106×g) for 30 mins and collected for experiments.

For SE NP coatings, 2-hole 1.5 mm titanium plates and screws (kindly provided by Medartis) were placed in a beaker to which a sodium selenite solution was added and followed by an ascorbic acid solution at a volume ratio of 3:40 (sodium selenite:ascorbic acid) and gently mixed. Se NPs formed within ca. 60 mins and decorated the plates and screws which were then rinsed 3 times in purified water.

### Material characterization

The particles were imaged using transmission electron microscopy (TEM). For this, a drop of particle suspension was placed on a TEM grid and imaged with a FEI Tecnai TF20 transmission electron microscope operating at 200 keV. Particle size and zeta potential were investigated using a zeta sizer (Nano ZS, Malvern Instruments, Malvern, UK).

The chemistry of the particles was studied using X-ray photoelectron spectroscopy (XPS, VG ESCALAB 220i-XL, VG Scientific, Sussex, UK) equipped with a monochromatic Al Kα X-Ray source, which emitted photon energy of 1,486.6 eV at 10 kV and 12 mA. Spectra were obtained at a step size of 0.1 eV (region/high resolution scans).

Uncoated and NP-coated plates and screws were imaged using scanning electron microscopy (Quanta ESEM) without conductive coatings.

### In vitro antimicrobial tests

MRSA (ATCC 43300) and MRSE (ATCC 35984) were kindly provided by Dr. Jonathan Wilksch from the Department of Microbiology and Immunology, The University of Melbourne (Victoria, Australia). Bacteria were cultured overnight in brain–heart infusion broth (BHI broth, BD Biosciences, North Ryde, NSW, Australia) in an aerobic incubator at 37°C. At late log phase (OD650~0.8), a 1:100 dilution of the bacterial culture in 0.9% saline (v/v) was counted using a flow cytometer (Cell Lab Quanta, Backman Coulter, Mount Waverley, Australia ) in the presence of 1 µL Syto 9 and 1 µL propidium iodide (PI) to every 1 mL of saline. Syto 9 and PI were prepared according to the manufacturer’s recommendations (LIVE/DEAD BacLight Bacterial Viability and Counting Kit for flow cytometry; Invitrogen, Thermo Fisher, Waltham, MA, USA). Finally, the bacterial culture was diluted to a density of 2.5×10^6^ cells/mL and 100 µL of this bacterial suspension was inoculated together with 100 µL of the NP suspension in a 96-well plate under aerobic condition at 37°C. The growth of bacteria in the plate was monitored overnight by measuring the OD of the bacterial suspension at 620 nm. Another 96-well plate was prepared as above and used for a live/dead assay after 4 hrs of NP treatment using a flow cytometer, as described above.

Experiments were conducted in duplicate and repeated at least three times unless otherwise indicated. Results were reported as the mean ± SDs.

### In vitro cell biocompatibility

Ti and Ti-Se samples were sterilized by autoclaving prior to cell seeding.

#### Cell seeding

Primary human osteoprogenitor cells (hOBs) were isolated from the knee of a male donor undergoing a knee replacement as described previously and approved by the Human Research Ethics Committee (HREC/14/QPCH/186, QUT/1400001024). Osteoprogenitor cells were grown in growth media (GM), containing minimum essential medium eagle - Alpha M (αMEM), supplemented with FBS and 1% penicillin/streptomycin (all from Thermo Fisher Scientific), and used at passage 5 for seeding on the constructs. Briefly, 24-well plates were coated with an autoclaved 1% Agar solution (Sigma-Aldrich) and let to set, to provide non-adhesive surfaces. Sterilized Ti/Ti-Se constructs were placed in the center of each well. Cell solutions were prepared at an average concentration of 1×10^5^ cells, and 500 µL of the cell suspensions was added to each well. Well-plates were transferred onto a rocking mixer platform (RPM4, Ratek Laboratory Equipment) in a humidified incubator (37ºC, 95% air, 5% CO_2_) overnight. Next, the suspensions were aspirated and the constructs were washed with fresh PBS prior to being transferred to a fresh 24-well plate topped-up with GM. Media was changed every 3 days and co-cultures lasted 7 days in total.

#### Metabolic activity

Metabolic activity was measured using the PrestoBlue cell viability assay (Invitrogen, Australia) at day 1, 4, and 7 of co-culture. Ti and Ti-Se constructs in co-culture with primary hOBs were incubated with GM, containing 10% v/v PrestoBlue of the cell viability assay for 2 hrs in a humidified incubator (37ºC, 95% air, 5% CO_2_). Cell-free constructs were used as negative controls. Upon incubation, the solutions were transferred into black 96-well plates (Corning, Mulgrave, VIC, Australia). Fluorescence (excitation 544 nm, emission 590 nm) was determined using a FLUOstar Omega plate reader (BMG LABTECH, Mornington VIC, Australia) and corrected with a negative control background. Mean ± SEs are presented with n=6/group.

#### Cell morphology

Ti and Ti-Se constructs were collected after 1 and 7 days of co-culture. Briefly, media was aspirated and the constructs were washed in PBS twice, before fixation in 4% paraformaldehyde (PFA, Sigma-Aldrich) for 12 mins at room temperature. The constructs were then incubated with Fluorescein isothiocyanate  (FITC)-conjugated phalloidin (200 U/mL) and DAPI (5 µg/mL) in 0.5% BSA/PBS for 1 hr. After three washes in PBS (10 mins each), fresh PBS was added. Imaging was done using a Nikon spectral spinning disc confocal microscope (SDC, X-1 Yokogawa spinning disc with Borealis modification) fitted with a Plan Apo 10×objective. Fluorescence was recorded in the green (ex 488 nm) and blue (ex 405 nm) channels for actin filaments (phalloidin) and cell nuclei (DAPI), respectively. Maximal intensity projections were made from z-stacks with 2 µm as the step size and thickness averaging 20 µm.

### Pilot in vivo antimicrobial trial

#### Animal surgery

The in vivo study was conducted at the Experimental Medical and Surgical Unit, St. Vincent’s Hospital. All experimental protocols were approved by the Animal Ethics Committee (AEC) of St. Vincent’s Hospital, Melbourne, Australia. The methods were carried out in accordance with the guidelines and regulations from the Australian National Health and Medical Research Council guidelines for the care and use of laboratory animals. Sprague-Dawley rats were anesthetized using ketamine 75 mg/kg with xylazine 10 mg/kg IP then placed on isoflurane 1–2% to maintain anesthesia. Rats were also injected with carprofen 5 mg/kg SC as an analgesic. The legs of the rats were shaved and then decontaminated using chlorhexidine. A small longitudinal incision of approximately 2 cm was made allowing a lateral approach to one femur. The bone was exposed and a surgical titanium 2-hole plate with or without Se NP coating was applied to the bone, using a dental drill to provide guide holes for the screws, which were correspondingly with or without the Se NP coating. Bacterial suspensions of the selected test doses (total 10^5^ colony-forming unit (CFU) for MRSE and 10^2^ CFU for MRSA) were injected in 100 µL of sterile 0.9% saline on top of the plate. These bacterial doses were determined for each of the bacterial species via an initial dose screening study as being sufficient to reliably form suitable biofilms in this animal model (data not shown). Control animals received plates with 100 µL of saline injected on top of the plates. The internal fascia was then closed with 4/0 silk sutures and the skin closed with absorbable sutures and clips. The operation and treatment was then repeated on the second femur.

The rats were then monitored until they recovered from the anesthetic and rehydrated with 5–10 mL warm lactated Ringers' solution if required. After monitoring for 3 hrs, the rats were placed in separate cages and returned to group housing after 48 hrs to prevent interference with surgical sites. Four weeks after implantation, the rats were euthanized using IV injection of Lethobarb (0.25 mL), and the implanted plates and screws were surgically removed to be assessed for biofilm burden.

#### Biofilm analysis

One plate from each animal was fixed in 4% glutaraldehyde followed by dehydration in ethanol and treated with 100% hexamethyldisilazane for 30 mins for scanning electron microscopy imaging of biofilm with a gold conductive coating. The other plate was fixed in 4% PFA for 1 hr followed by immunostaining for bacteria. For this, rabbit anti-MRSA (Ray biotech, Peachtree Corners, GA, USA) and mouse anti-*Staph epidermidis* (Acris Antibodies) were used to recognize MRSA and MRSE on the plates. Appropriate secondary antibody fluorescent conjugates (goat anti-rabbit IgG conjugated to Alexa Fluor 555 and rabbit anti-mouse IgG conjugated to Cy3, both from Molecular Probes, Scoresby, VIC, Australia) were used to bind to the primary antibodies following the manufacturer’s instructions. After staining, the plates were imaged with a Nikon A1R confocal microscope using a 40X objective lens. Images were obtained at 512×512 resolution and z-stacks created with a 1 μm step size. These were then converted to 3D reconstructions so that the full biofilm thickness could be visualized and imaged.

Extracted screws were rinsed three times with sterile saline to remove any free bacteria not associated with biofilms, followed by brief (~30 seconds) vortexing and sonicating to retrieve biofilm bacteria from the biofilm. The bacterial suspensions were serially diluted, then plated on blood agar, and incubated at 37°C overnight to count the number of CFU.

The wound pockets were also swabbed with sterile cotton swabs to assess the number of CFU in the surrounding tissue. The swabs were placed in sterile saline and sonicated for 30 s to retrieve the bacteria before plating on blood agar and incubating at 37°C overnight to determine the number of CFU.

## Results

### NP synthesis and characterization

**Figure 1:**
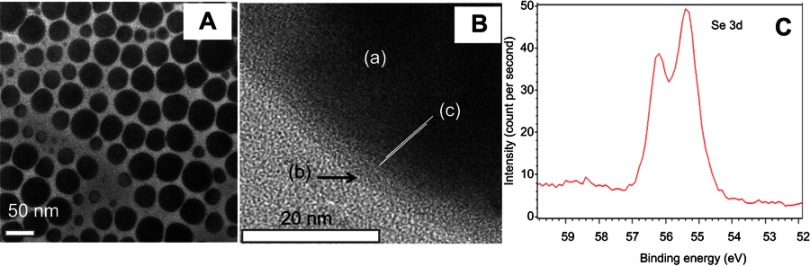
Characterization of Se nanoparticles. TEM imaging (**A**) shows a near-spherical shape of the nanoparticles with major sizes about 30–70 nm. High-resolution TEM (**B**) shows the stabilizing PVA layer (b) adsorbed on the core Se (a) which appears to be crystalline [c] showing the direction of crystal planes). (**C**) XPS analysis showing Se 3d peaks at 55.4 and 56.2, suggesting its zero oxidation state.

The synthesis of these Se NPs has been reported before.^32^,^33^ Briefly, our group has characterized this material extensively to show spherical particles of hydrodynamic diameters ranging from 50 nm to 200 nm (using dynamic light scattering techniques and TEM – Figure 1). The particles were also confirmed to have zero oxidation state (as indicated from the location of Se 3d peak at 55.2 eV by XPS) and exhibited negative surface charges as indicated by a zeta potential of approximately −24 mV.

### In vitro antimicrobial properties

**Figure 2:**
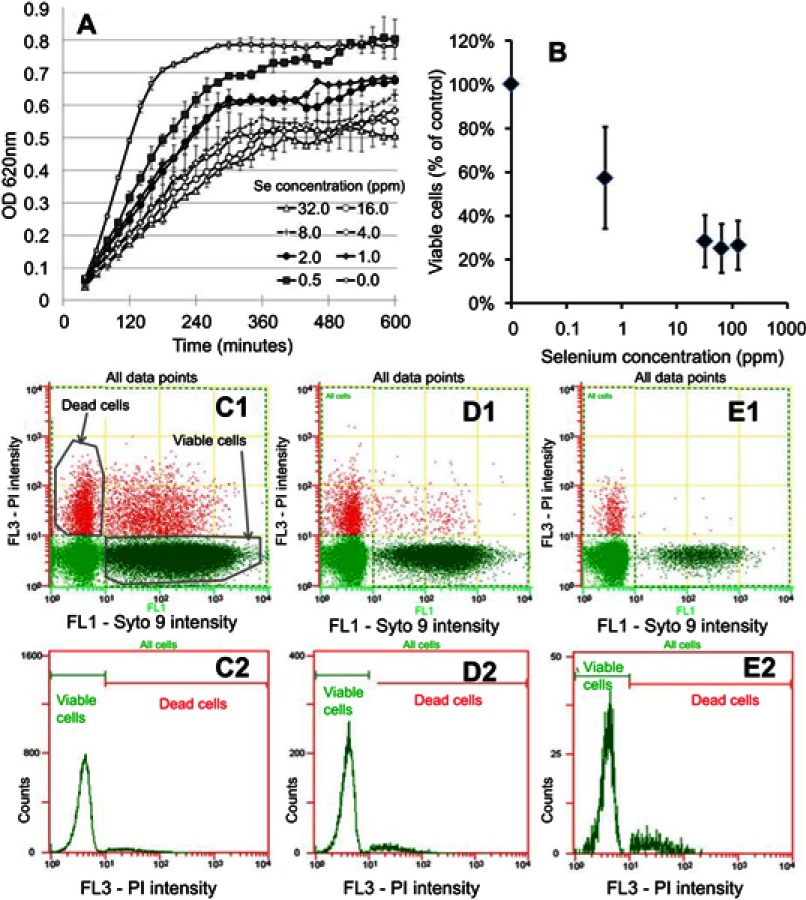
In vitro testing results against MRSA. (**A**) OD growth curves of bacteria solutions treated with increasing amount of Se from 0.25 ppm to 128 ppm showing reduced growth of bacteria treated with the Se NPs. (**B**) Live/dead assay results showing reduced live cell numbers when bacteria were treated with increasing concentrations of Se NPs. (**C1, D1,** and **E1**) Representative flow cytometry 2D dot plots of bacteria, treated with 0 ppm Se (ie, untreated), 0.5 ppm Se and 128 ppm Se and corresponding histograms (**C2, D2,** and **E2**).

The Se NPs were first tested against MRSA. In the presence of the NPs, bacterial growth was slowed, as indicated by the decrease in the slope of the growth curves with increasing Se concentration (Figure 2A).

This trend was further confirmed using flow cytometry to determine the number of viable cells (Figure 2B), which clearly showed significant decreases when the bacteria were treated with Se NPs. This decrease was observed at a Se concentration as low as 0.5 ppm. Higher Se concentrations resulted in further decreases in viable cell numbers; however, there was no significance change in viable cell numbers among concentrations above 32 ppm.

**Figure 3:**
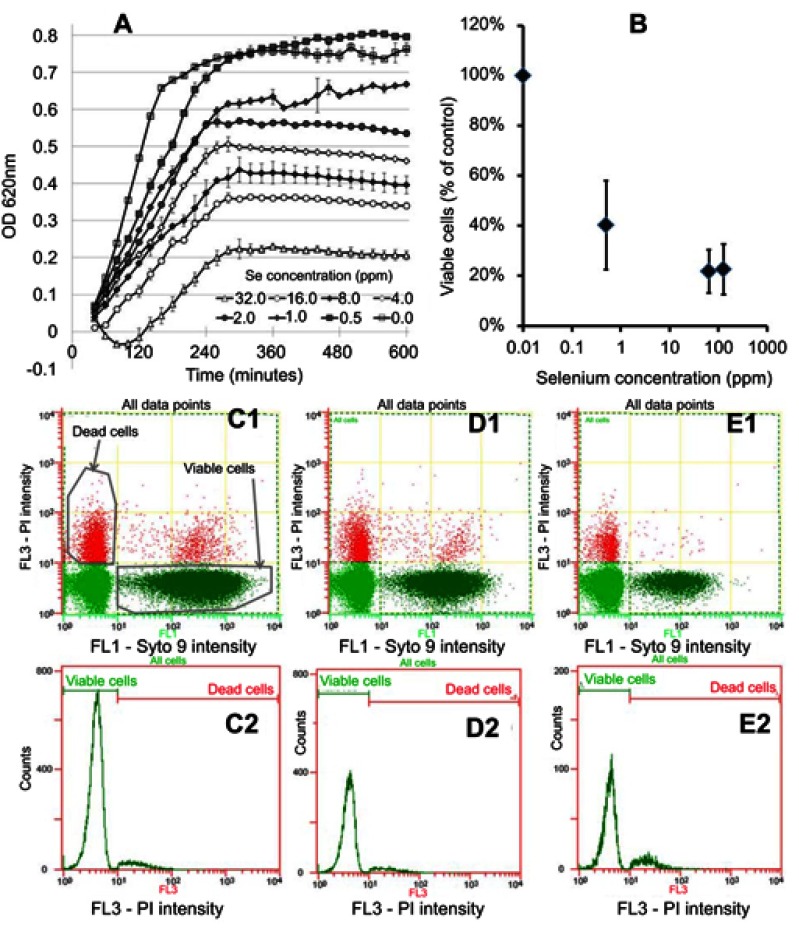
In vitro testing results against MRSE. (**A**) OD growth curves of bacteria solutions treated with increasing amounts of Se NPs from 0.25 ppm to 256 ppm showing reduced growth when bacteria were treated with Se. (**B**) Live/dead assay results showing reduced live cell numbers when bacteria were treated with increasing concentrations of Se NPs. (**C1, D1,** and **E1**) Flow cytometry 2D dot plots of bacteria, treated with 0 ppm Se (ie, untreated), 0.5 ppm Se and 128 ppm Se and corresponding histograms (**C2**), (**D2**), and (**E2**).

In separate experiments, MRSE which is an important drug-resistant bacterium causing device-associated infections was treated with Se NPs. Se NPs were found to inhibit the growth of MRSE, as indicated by decreases in the slopes of the growth curves when the bacteria were treated with the NPs (Figure 3A). The data were processed by subtracting the value for blank control (no bacteria). The measurement has a random error of approximately 0.05, thus making some subtracted values dropping below zero. This inhibition was further confirmed by analysis of the numbers of viable cells (Figure 3B), which showed a reduction in cell numbers to concentrations as low as 0.5 ppm Se.

The decrease in viable cell concentrations when treated with selenium was not found to be linked with a significant reduction in dead cells (Figures 2 and 3 – C1, D1, and E1); rather it was associated with a significant reduction in the total number of cells, indicating the inhibitory effects of selenium.

### Selenium coating applied on Ti substrates and bioactivity testing

#### NP coating of titanium plates and screws and response of osteoblasts in vitro

**Figure 4:**
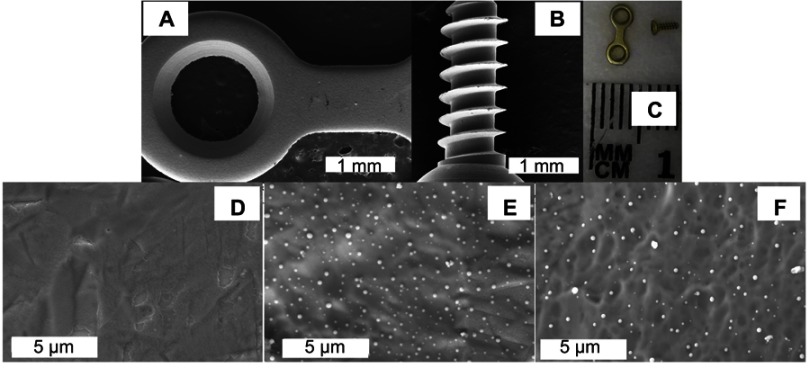
Representative images of the titanium plates and screws used in in vivo experiments. (**A** and **B**) are low-magnification SEM images of a coated plate and screw, respectively. (**C**) is a photograph of an uncoated plate and screw. Coated plates and screws showed the same gross appearance and microscopically smooth surface (**A** and **B**). (**E** and **F**) are SEM images of coated plate and coated screw surfaces, respectively, showing the clear presence of Se nanoparticles compared to an uncoated surface (**D**).

The same reduction chemistry used for making NPs in suspension was used to create NP coatings on the plates and screws. This method has been successfully used previously to create Se NP coatings on metallic and polymeric substrates and the coatings were found strongly adhered.^32^,^39^ For the purpose of the pilot study, the plates and screws were coated with Se NPs using conditions that were used before to coat titanium substrates at a density of approximately 6×10^6^ particles/mm^2^ (measured using Image J analysis of SEM images) and were shown previously to be non-toxic to osteoblast cells.^39^ This condition was also chosen because it was shown before that this density was sufficient to significantly inhibit the growth of *S. aureus* on Se-coated polymeric surfaces.^32^ The particles had spherical shapes, with sizes ranging from approximately 30 to 70 nm in diameter and uniformly decorated the substrate surfaces (Figure 4).

**Figure 5:**
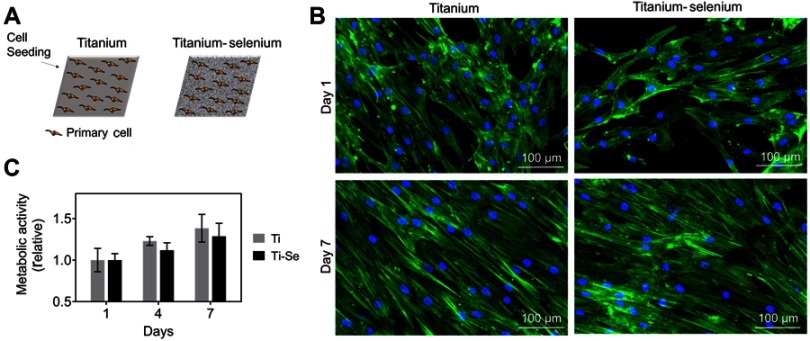
In vitro biocompatibility results of osteoblasts cultured on Ti and Se-coated Ti substrates. (**A**) Schematic of titanium (Ti) and titanium-selenium (Ti-Se) plates being seeded with human primary osteoprogenitor cells. (**B**) Metabolic activity over 7 days is similar between Ti and Ti-Se plates. (**C**) Morphology of primary osteoprogenitor cells after 1 and 7 days post seeding shows the typical elongated phenotypes and full coverage of the plates, without differences between substrates.

We first confirmed the non-toxicity of the SE NP coatings^31^–^33^,^39^,^40^ by direct culturing of primary osteoblast cells. hOBs were co-cultured on Ti and Ti-Se plates up to 7 days, to assess morphology and metabolic activity (Figure 5A). The hOB cells attached to both Ti and Ti-Se substrates had similar morphologies after 1 day of culture (Figure 5C).

After 7 days, both substrates were homogeneously coated by hOBs with elongated morphologies typical for primary hOB cells, with no differences between Ti and Ti-Se.

Metabolic activity was also shown to be similar between both constructs over time (Figure 5B). In conclusion, the presence of Se NPs did not alter in vitro cell response compared to uncoated titanium plates.

#### In vivo biofilm and bacteria analysis

**Figure 6:**
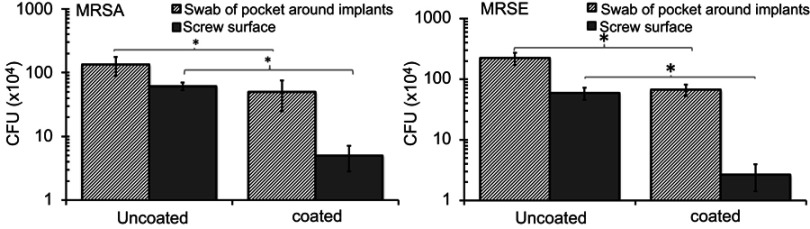
Numbers of colony forming units (CFU) retrieved from wound pocket swabs and screws 4 weeks after implantation showing decreased CFU counts with the Se NP coating. Data = mean ± SEM (n=3); * *p*<0.05.

First, we examined the effects of Se coatings on the numbers of bacteria in the tissue surrounding the implants and adherent to the screws. The numbers of CFU retrieved from swabs sampling the tissue surrounding uncoated implants were higher than those from the tissue surrounding the coated implants (Figure 6; p=0.03). The numbers of CFU retrieved from the screws also showed a similar trend (Figure 6).

**Figure 7:**
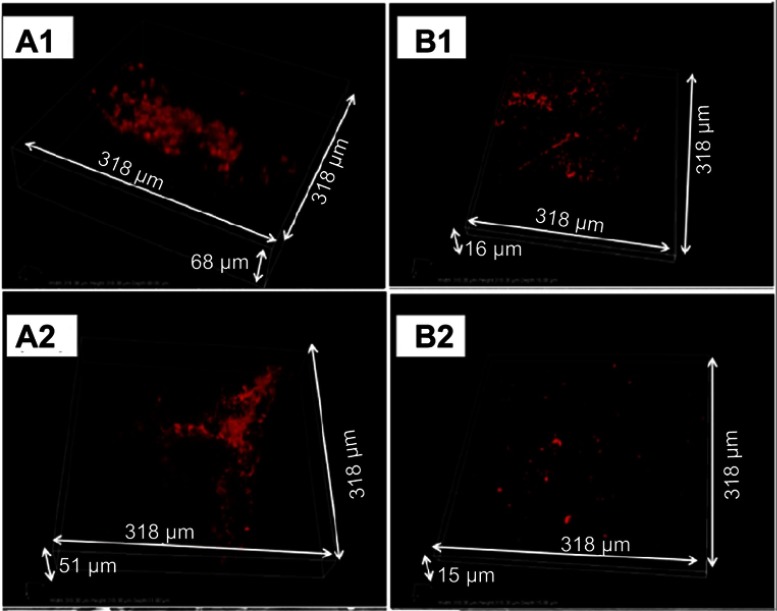
In vivo biofilm formation by MRSA (**A1** and **B1**) and MRSE (**A2** and **B2**) on implanted plates. Representative confocal microscopy snapshots of 3D reconstructions (obtained with a 40X objective) of immunostained bacteria on uncoated (**A1** and **A2**) and coated (**B1** and **B2**) plates after 4 weeks of implantation.

Biofilm formation was next evaluated on the plates which had flat surfaces that allowed confocal microscopy to be used more effectively than on the screws. Confocal microscopy imaging of the bacteria within the biofilms showed thick and dense layers on the uncoated plates compared to more individual, separated bacteria and bacterial aggregates on the coated plates (Figure 7A1, B1, A2, B2).

The aggregates on the coated plates had a maximal thickness of around 3 µm, while the biofilms on uncoated plates have maximal thickness ranging from 5 µm to 16 µm, confirming the multi-layer structure of these biofilms.

## Discussion

Despite significant improvement in surgical hygiene techniques and the use of pre-emptive antibiotics, bacterial infections remain a significant problem for medical devices such orthopedic implants. Opportunistic bacteria adhere irreversibly to a device’s surface and develop to form a polysaccharide biofilm protecting the embedded bacteria from antibiotics and host defense mechanisms.^11^,^41^ As a result, device-associated infections remain serious complications, with rates ranging from about 2% for hip-joint prostheses,^42^,^43^ and higher in traumatic injuries in contaminated environments.^44^,^45^ Prevention of bacteria colonization and biofilm formation on implant surfaces has, thus, been recognized as crucial because infected devices often necessitate device removal and revision surgeries which are costly (eg, approximately $50,000 for a hip-joint revision^46^), leading to prolonged hospital stay and increased treatment costs.

Surface topography, charges, or coatings have been shown to be able to reduce bacteria adhesion or kill bacteria. Surface nanotopography has been shown to reduce bacteria attachment and even cause membrane damage for adherent bacteria.^47^,^48^ Surface charges and hydrophilicity have also been shown to influence bacteria adhesion.^49^,^50^ Polymer brushes that repel bacteria (such as PEO, polyethylene glycol (PEG), etc.) or kill bacteria in contact (such as polycationic^51^ or NO-releasing coatings^52^) have also been extensively studied. Yet, these treatments either lack or have very short efficacy (from several hours to few days),^53^ are not mechanically stable, are toxic,^50^ or require complicated synthesis methods and equipment to apply on devices.^25^,^30^ Immobilization of antibiotics or antimicrobial peptides has also been investigated, yet they suffer from toxicity to mammalian cells^54^ and loss of activity.^55^

Inorganic NPs (such as selenium) are an advantageous platform for the design of the next generation of antimicrobial coatings as they have the capacity to kill microbes by multiple mechanisms, are stable, and can be readily immobilized onto various surfaces. These NPs can involve the production of ROS causing damage to various cellular components; disruption of microbial cell membranes; interruption of transmembrane transport processes; DNA damage; interruption of energy transduction; and inhibition of enzyme activity.^56^,^57^ Because of their inorganic nature, they are highly stable compared to other antimicrobial agents such as antibiotics and antimicrobial peptides. The inorganic NPs can also readily immobilize onto surfaces without losing activities.

We have significant experience with Se NP synthesis, immobilization, and testing of their antimicrobial activities.^31^-^33^,^39^,^40^ In the current work, we focused on their activities against two drug-resistant bacteria of significant importance in orthopedic implant infections, MRSA and MRSE, and evaluated their efficacy in an infected rat femur model.

We first evaluated the dose–response of the Se NPs in suspension form to establish their activity profiles. An aqueous-based method was developed to synthesize Se NPs. This synthesis method is based on the reduction of selenite salt in the presence of a stabilizing agent (for the production of NP suspensions) or a surface (for the production of NP coatings). The Se NPs were shown to have a zero oxidation state which is important because this form of Se is less toxic and less soluble than other Se forms such as selenate, selenite, and organic Se products.^35^–^38^ Our laboratory also demonstrated that these NPs had very low toxicity against red blood cells and embryonic fibroblast cells.^40^ For both MRSA and MRSE, flow cytometry data (Figures 2C1–E2 and 3C1–E2) showed that the decrease in bacterial cell viability seen in Figure 2A and B and Figure 3A and B can be attributed to a reduction in bacterial cell numbers. Several studies have indicated that metal NPs formed from Au, Ag, and Ag/Cu induced rapid cell lysis via membrane damage.^58^–^60^ Further, we have previously shown that Se NP did induce extensive membrane damage in MRSA.^31^ Thus, the loss of bacterial cell numbers in our study corroborates these findings that Se NPs would induce rapid cell lysis by extensively damaging the bacteria membrane of MRSA and MRSE.

Next the NPs were immobilized on Ti implants to form antimicrobial coatings using the same aqueous-based electroless deposition method. This method of coating is versatile and has been applied to a range of both metallic and polymeric surfaces in our laboratories. In the case of Se NPs in suspension, PVA was used as a stabilizing agent to provide steric hindrance and stabilize them. In the coating formulation, the NPs were immobilized on the Ti surface, and hence did not require a stabilizing agent. PVA itself does not have anti-bacterial activity as is well known in the literature and has no net charge; thus, it would not make any difference on the material’s antibacterial activity.

The Se NPcoatings were shown to slowly release soluble Se species which inhibit the growth of *S. aureus* through mechanisms related to free intracellular thiol depletion.^61^ The nanotopography created by the Se NPs was also previously shown to promote bone-forming osteoblast cell functions through increased select protein adsorption on the nanorough surfaces. In the current work, we focused on investigating this coating particularly for inhibiting orthopedic fixation infections associated with drug-resistant bacteria. The Ti plates and screws were applied to rat femurs and were challenged with MRSA or MRSE to simulate device-associated infections. The Se NP coatings were able to inhibit biofilm formation (Figure 6) and associated local contamination (Figure 5) following inoculation of implants with biofilm forming doses of bacteria. The reduction in local contamination was likely due to the in vivo release of selenium, although this needs to be further studied.^39^ It could also be likely that the swabbing process was not entirely consistent among the samples. The collection of tissue followed by homogenization and plating out to enumerate CFU (normalized by tissue weight) would be a more standardized ways to assess local contamination and will be followed in the future.

In a summary, this study examined the antimicrobial activity of Se NPs and their coatings against two important drug-resistant bacteria, MRSA and MRSE, in vitro and in vivo. The NPs were prepared by a simple aqueous-based redox synthesis method and showed in vitro antimicrobial activity at concentrations as low as 0.5 ppm. NP coatings were applied to titanium screws and plates and tested in an in vivo rat femur model. The results from this animal study showed that the coatings strongly inhibited biofilm formation on the implants and reduced the number of viable bacteria in the surrounding tissue. This study shows a proof of concept for a Se NP coatings as a potential anti-infective barrier for orthopedic medical devices in the setting of contamination with multi-resistant bacteria. It also represents one of the few (if only) in vivo assessment of SE NP coatings on reducing antibiotic resistant orthopedic implant infections.
